# Hybridization and low-light adaptability in California eelgrass (*Zostera* spp.)

**DOI:** 10.1038/s41477-025-02142-2

**Published:** 2025-10-29

**Authors:** Malia L. Moore, Nicholas Allsing, Nolan T. Hartwick, Allen Mamerto, Emily R. Murray, Rilee D. Sanders, Todd P. Michael

**Affiliations:** 1 Scripps Institution of Oceanography, La Jolla, CA USA; 2 Plant Molecular and Cellular Laboratory, Salk Institute for Biological Studies, La Jolla, CA USA; 3 Paua Marine Research Group, Long Beach, CA USA; 4 Department of Science and Conservation, San Diego Botanical Garden, Encinitas, CA USA; 5 Department of Cell and Developmental Biology, School of Biological Sciences, University of California, San Diego, La Jolla, CA USA

**Keywords:** Light responses, Plant ecology, Comparative genomics

## Abstract

The temperate seagrass *Zostera marina* is a foundational marine species that provides critical habitat in bays and estuaries throughout the Northern Hemisphere. Human activities and climatic events have necessitated *Z. marina* restoration, for which high transplant mortality rates call for innovative cross-disciplinary solutions. We identify a hybrid between *Z. marina* and *Z. pacifica* and explore the hybrid population as a tool for restoration from a genomic perspective. Among several habitat distinctions, *Z. pacifica*, an endemic to the Southern California Bight (West Coast, USA), is deeper-living and may encode resilience to low light, a leading factor of seagrass restoration failure. We construct a haplotype-resolved chromosome-scale genome assembly of the hybrid and several California *Zostera* accessions to describe the divergence between *Z. marina* and *Z. pacifica* and characterize the hybrid’s stage of maturity as an F_1_. Transcriptomes of *Z. marina* and the hybrid subjected to reduced light in an experimental mesocosm reveal divergent trends in photosynthesis, carbohydrate utilization and stress responses. Photoperiod regulation by *Z. pacifica* orthologues of key circadian clock genes, prominently *LATE ELONGATED HYPOCOTYL* and *WITH NO-LYSINE KINASE*s, may drive this response. By describing the hybridization event using genomic and transcriptomic methods, this study presents preliminary evidence of low-light tolerance modulated by a labile circadian clock to motivate further ecological and functional studies of this hybrid as an experimental tool to access *Z. pacifica* genetics and its relevance to restoration.

## Abstract (web summary)

Seagrass genomes of the deep-water *Zostera pacifica* and shallow-water *Z. marina* from the Eastern Pacific Ocean provide evidence for low-light adaptation exemplified in the wild hybrid *Z. marina* × *pacifica* subjected to low-light stress in aquaria.

## Main

Seagrasses are marine plants that form the foundation of productive and ecologically valuable coastal ecosystems, promoting carbon-rich sediment deposition and retention^1,2^. Common eelgrass (*Zostera marina*) is the dominant seagrass in bays and estuaries along the West Coast of North America. In California, *Z. marina* inhabits estuaries and bays from intertidal to subtidal depths of 4 m, forming extensive and structurally complex habitats that provide critical ecosystem services such as cycling nutrients, improving water quality and stabilizing sediment to reduce coastal erosion^3–5^. As an ecosystem engineer, *Zostera* spp. provide nearshore habitat for juvenile fish and invertebrates, which is designated at the federal level as an essential fish habitat^3^. These vital systems are, however, experiencing large-scale losses from anthropogenic damage^6^. Dredging, boating activity and coastal development have reshaped the bays that eelgrass populates^7^. Changes in light, temperature and pollutants threaten the photosynthetic capacity of seagrasses and make them more prone to disease^8,9^.

Eutrophication-induced light limitation has emerged as a leading physiological cause of seagrass die-offs and restoration failures in California bay systems^10^. When subjected to transient light reduction in the marine environment, eelgrasses metabolize non-structural carbohydrates (NSCs) stored in the rhizome^8,11–14^. These NSCs consist of ~87% sucrose, lending *Z. marina* the nickname ‘marine sugarcane’ and providing the plant with a metabolic fallback plan when light falls below the light compensation point, at which carbon usage outstrips carbon assimilation^11^. This stress response has been demonstrated in the field and in aquaria and quantified with direct measurements of sucrose and starch as well as gene expression in the leaf^11–14^. Chronic and episodic light disturbance in eelgrass beds during their growth season reduces survivorship of eelgrass by depleting NSCs prior to overwintering^8^. Following the sequencing of the *Z. marina* genome, mechanistic study of the eelgrass low-light response has focused on NSC depletion, with genes identified for sucrose transport and stress signalling in the abscisic acid (ABA) pathway^13,15,16^. Yet, low-light tolerance varies seasonally and among populations in *Zostera*, and it remains to be seen how this resilience is genetically determined^17^.

The open-coast, offshore eelgrass *Z. pacifica* thrives at over triple the depth of *Z. marina*, making it an interesting case study in eelgrass low-light tolerance. Endemic to Southern California, *Z. pacifica* has a distinctive wide-leaf phenotype and principally inhabits sandy-sediment areas in exposed coastal shelf habitats and offshore islands in depths ranging from 6 to 22 m (refs. ^18–20^). Foundational studies of eelgrass light requirements agree that photosynthetically active radiation (PAR) of 3–3.8 mol m^−2^ d^−1^ is the minimum for *Z. marina* survivorship, while maximum growth takes place at 15–20 mol m^−2^ d^−1^ (refs. ^21–24^). Offshore *Z. pacifica* beds were found to grow at the low range of *Zostera* light requirements, averaging 3–4 mol m^−2^ d^−1^, with several transient and seasonal storm periods <1 mol m^−2^ d^−1^ (ref. ^20^). In addition to light quality, the *Z. pacifica* environment differs in temperature, pressure, dissolved oxygen content, sediment composition and wave activity from the estuaries and bays that *Z. marina* most commonly inhabits^20^.

*Z. pacifica* will hybridize with *Z. marina* in bays. Observed populations intermediate to *Z. marina* and *Z. pacifica* in San Diego Bay and Newport Bay suggest that hybridization is a consequence of *Z. pacifica* restoration transplants^18^. Our surveys located a persistent hybrid eelgrass bed in Mission Bay’s Mariner’s Basin in San Diego County at 2–4 m depth. At this site in October 2011, the US Army Corps of Engineers contracted eelgrass restoration to compensate for dredging impacts, a practice known as mitigation^25,26^. This project included a 1.39-acre mixed *Z. marina* and *Z. pacifica* transplant intended to address suspected light limitation, given the depth and bathymetry^25^. The mixed-species site successfully restored eelgrass density, though post-transplant surveys do not resolve whether success stemmed from site conditions, the presence of *Z. pacifica* or that of a hybrid^26^. Currently, the site hosts a robust mix of *Z. marina* and hybrid eelgrass.

The Mariner’s Basin eelgrass hybrid *Z. marina* × *pacifica* (hereafter referred to as the hybrid) represents a striking intermediate of natural and human-facilitated hybridization. Both species are native to the region, but they have high niche differentiation, making opportunities for introgression rare^18,19^. Other marine plants, including the euryhaline grass *Ruppia* and the seagrass *Posidonia*, have been shown to hybridize within their native ranges^27,28^. Interspecific hybridization increases genetic diversity and may produce hybrid vigour (heterosis) or adaptive radiation within the mosaic habitat pressures of a hybrid zone^29,30^. Anthropogenic threats to eelgrass necessitate creative solutions to restoration. With both deep- and shallow- living parentage, the hybrid may colonize intermediate depths in bay environments where *Z. marina* is ephemeral or absent, or may better withstand seasonal storms. Here we describe the hybridization using genomic and transcriptomic methods and present preliminary evidence of low-light tolerance to motivate further ecological and genetic studies of this hybrid.

## Results

### Identifying and sequencing a *Zostera* hybrid

**Fig. 1:**
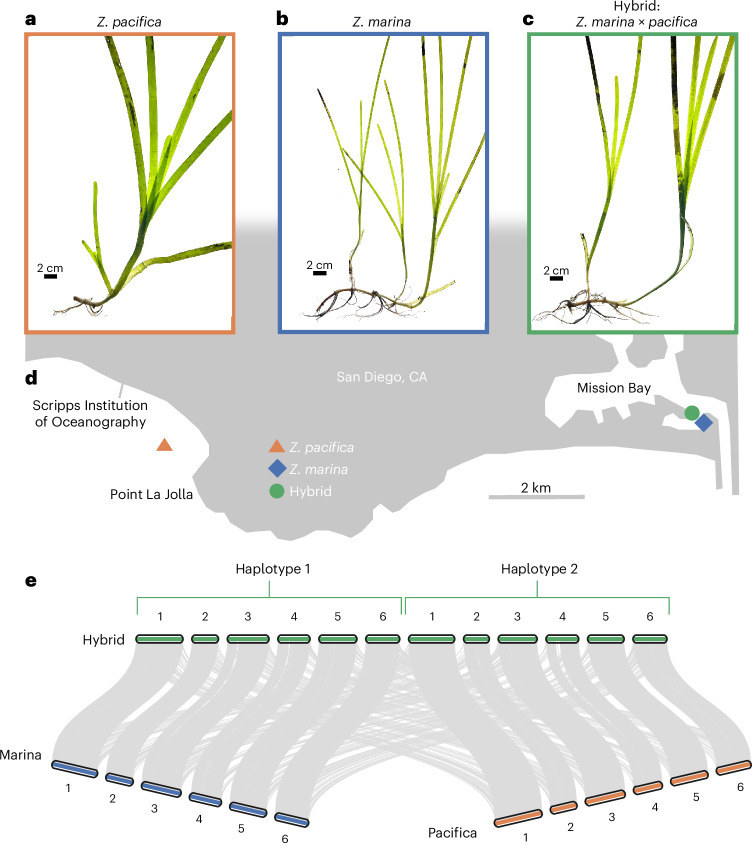
Leaf width phenotype differentiates *Zostera* species and identifies a hybrid. **a**–**c**, The wide-leaved *Z. pacifica* (Zpac1022-Sio2) (**a**) versus the narrow-leaved Southern California *Z. marina* (Zmar913-Mar3) (**b**) and the hybrid (Zmar912-Mar1) with intermediate leaf width (**c**). **d**, The hybrid and proximal *Z. marina* and *Z. pacifica* were collected in Mission Bay and La Jolla; for the sample metadata, see Supplementary Table 1. **e**, De novo genome assemblies for the hybrid, *Z. marina* and *Z. pacifica* resolve the hybrid as a diploid F_1_, with haplotype assemblies referenced as Hap1-*marina* and Hap2-*pacifica*.

**Extended Data Fig. 1:**
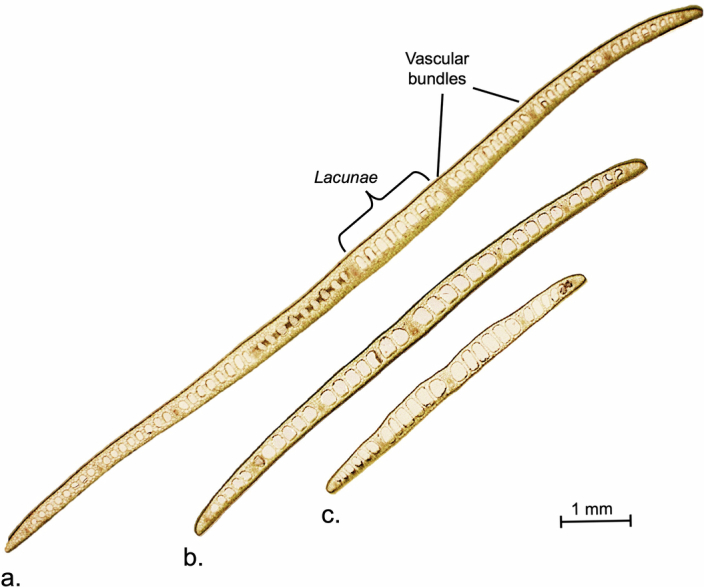
Leaf cross-sections. Mature leaf tissue from **(a)** *Z. pacifica* **(b)** Hybrid **(c)** *Z. marina* imaged with a Zeiss dissecting microscope. Vascular bundles (VB) alternate with a series of functionalized aerenchyma called lacunae, which are conspicuous in leaf cross-section and contain the air which creates *Zostera* leaf buoyancy. As leaf width increases, this VB-lacunae pattern repeats, with Southern California *Z. marina* having up to five VBs, Hybrid seven, and *Z. pacifica* nine, though these counts vary from young to mature tissue. Original images (no cropping or contrast enhancement) available as Supplementary Data 1.

**Extended Data Fig. 2:**
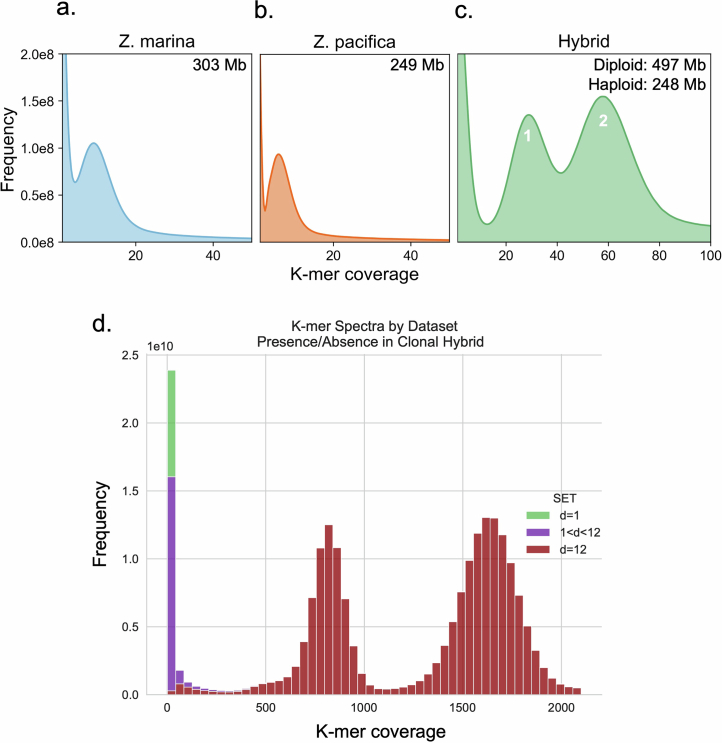
K-mer frequency estimates genome size and Hybrid clonality. Illumina short reads produce K-mer frequency histograms for **(a)** *Z. marina* accession Zmar913-Mar3, **(b)** *Z. pacifica* accession Zpac1022-Sio2 and **(c)** Hybrid accession Zmar1102-1A. Peak **1** indicates the heterozygous peak (K-mer coverage at max. frequency used to estimate diploid genome size) and **2** the homozygous peak (estimates haploid genome size). Twelve Hybrid plants from Mariner’s Basin **(d)** demonstrate homogenous K-mer content at the homozygous and heterozygous peaks, indicating that all individuals are clones. SET d = 1 indicates K-mers found in only one individual in the set, 1<d < 12 some shared individuals but not all, and d = 12 all individuals.

*Z. pacifica*’s wide leaves (Fig. 1a) contrast with the narrow leaves of Southern California *Z. marina* (Fig. 1b) and express as average leaf width in the hybrid (Fig. 1c). Physiologically, increased leaf width in eelgrass is produced by an increase in cell number rather than cell size (Extended Data Fig. 1a–c). In Mariner’s Basin, long, broad leaves distinguish hybrid eelgrass from the surrounding *Z. marina*. We collected nearby *Z. marina* (Zmar913) and *Z. pacifica* (Zpac1022) from the Matlahuayl State Marine Reserve in La Jolla (Fig. 1d,e). Through resequencing and counting *K*-mers, we estimated diploid genome sizes of 303 Mb for Zmar913, 249 Mb for Zpac1022 and 497 Mb for the hybrid, which displays much higher heterozygosity than either inbred (Extended Data Fig. 2a–c).

We constructed a haplotype-resolved genome of the hybrid, along with Zmar913 and Zpac1022 and several *Z. marina* accessions from broader California. The hybrid assembly is highly contiguous (with pre-scaffolding contig N50s of 25.7 Mb for haplotype 1 (Hap1) and 16.1 Mb for Hap2) and has high per-base accuracy (assembly quality values (QV) = 42.6 and 43.4) (Supplementary Table 1). All assemblies, excluding a lower-quality sample from Humboldt, achieved Benchmarking Universal Single-Copy Orthologs (BUSCO) completeness of 98.1–98.4%, on par with the *Z. marina* reference genome at 98.4%. We annotated protein-coding genes in each haplotype of the hybrid, hereafter referred to as Hap1-*marina* and Hap2-*pacifica*, and these annotations achieved 95.8% and 96.0% BUSCO completeness, respectively (Supplementary Table 1).

**Extended Data Fig. 3:**
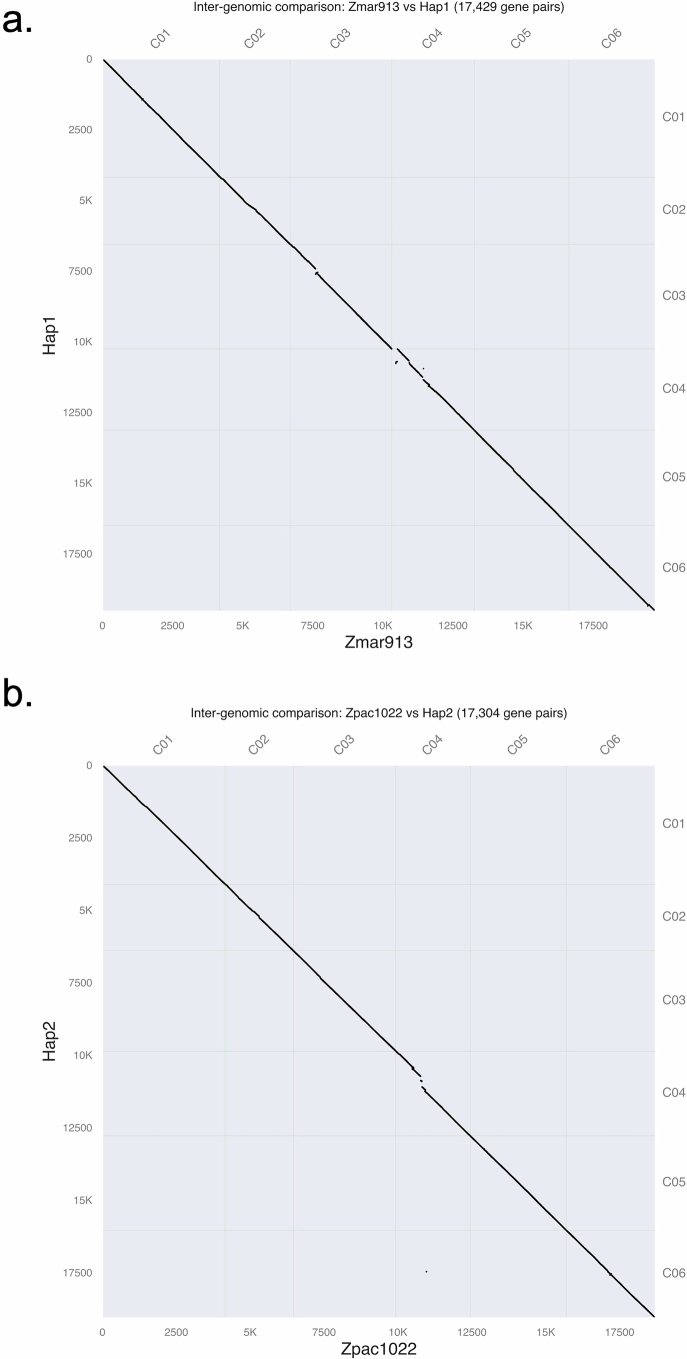
Syntenic ortholog dot plots of Hybrid and scaffolded *Z. marina* and *Z. pacifica* assemblies. Syntenic orthologs of **(a)** Zmar913-Mar3 vs Hybrid Hap1-*marina* and **(b)** Zpac1022-Sio2 vs Hybrid Hap2-*pacifica*. Numerical axes are in terms of syntenic gene count, presented in chromosomes C01-C06, not base pair position. Plots generated with JCVI Toolkit.

**Extended Data Fig. 4:**
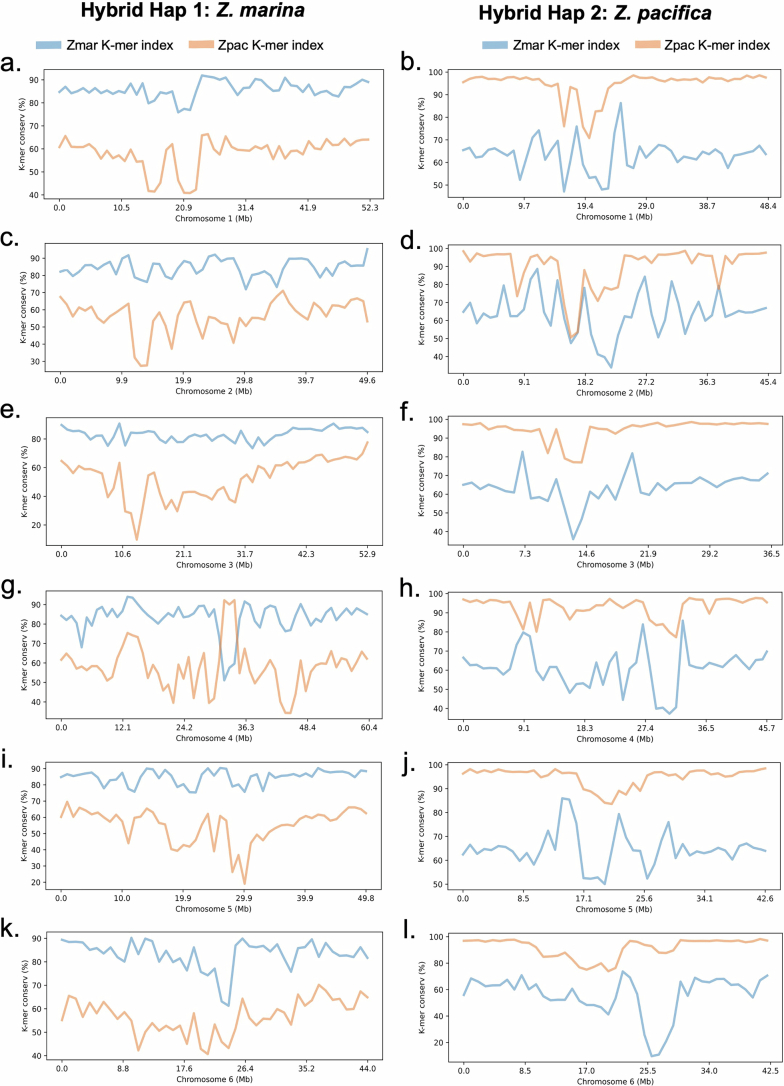
Hybrid is ‘F1’. (**a**-**l**) K-mer identity of all Hybrid scaffolded chromosomes (including non-coding regions) to *Z. marina* or *Z. pacifica*, visualized with PanKmer anchor plots.

By resequencing 12 hybrid individuals distributed throughout the site, we estimated that all hybrids displaying the characteristic wide leaf at the site are clones (Extended Data Fig. 2d). In the assembled hybrid genome, Hap1-*marina* aligns with Zmar913 and Hap2-*pacifica* with Zpac1022, with no conspicuous recombination of coding sequences (Fig. 1e). Hap1-*marina* shares 17,418 syntenic orthologues with Zmar913 (89.2% of on-scaffold genes), and Hap2-*pacifica* shares 17,304 with Zpac-1022 (90.7%) (Extended Data Fig. 3a,b). There are no apparent rearrangements between homologous chromosomes indicative of a more mature hybrid (Extended Data Fig. 4a–l), outside of a highly repetitive centromere sequence in chromosome 4 (Extended Data Fig. 4g), which is more likely the result of misassembly or scaffolding inaccuracies in this repeat-rich area of the genome.

### *Z. pacifica* speciation

**Extended Data Fig. 5:**
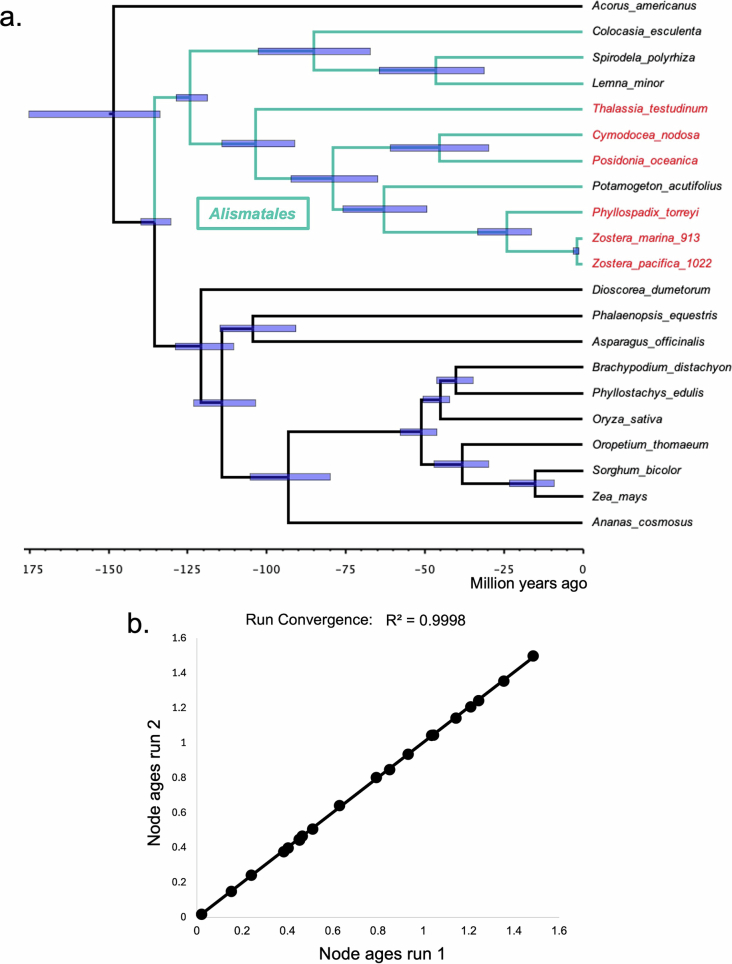
Phylogenetic reconstruction of seagrasses within the monocots. **(a)** Time-calibrated phylogeny with red text indicating seagrasses, with the *Alismatales* in turquoise. **(b)** Convergence of subsequent MCMCtree runs.

The Southern California Bight endemic *Z. pacifica* is considered a distinct species from *Z. marina* given differences in their ecology. This speciation had not been corroborated with genomic evidence beyond microsatellite phylogenetics and plastid assembly, which found the *Z. pacifica* chloroplast to be 99.4% similar to that of *Z. marina*^18,31^. We estimated that *Z. pacifica* and *Z. marina* diverged from a common ancestor 1.17 million years ago (Ma) using synonymous substitution rates, and we derived a range of 1.15–3.04 Ma using phylogenetic reconstruction of seagrasses within the monocots from multisequence alignment (Extended Data Fig. 5a,b and Supplementary Data 2).

**Fig. 2:**
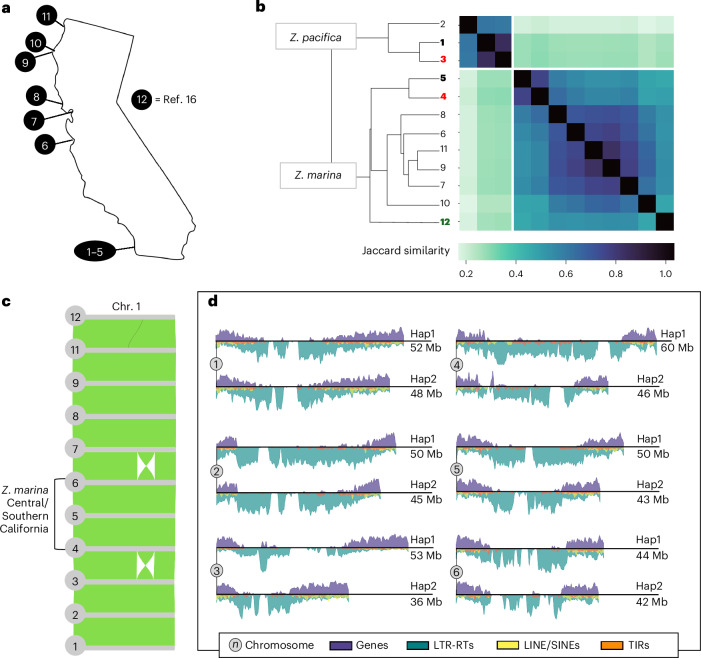
*Z. pacifica* divergence from *Z. marina* at the genome and pangenome scales. **a**, California eelgrass collections from south to north with latitudes: Mission Bay (32.8° N), Elkhorn Slough (36.8° N), San Francisco Bay (37.9° N), Tomales Bay (38.2° N), Eel River Estuary (40.6° N), Humboldt Bay (40.7° N) and Crescent City Harbor (41.7° N). The collection locations are numbered as follows: (1) Zpac1022-Sio2, (2) Zpac1022-Sio1, (3) hybrid Hap2-pacifica, (4) hybrid Hap1-*marina*, (5) Zmar913-Mar3, (6) Zmar1017-Elk1, (7) Zmar1015-Sfb1, (8) Zmar1010-Tom3, (9) Zmar106-Eel1, (10) Zmar107-Hum1, (11) Zmar108-Cre1 and (12) Zmar668 Finland^16^. Zpac, *Z. pacifica*; Zmar, *Z. marina*. The accession names correspond to those in Supplementary Table 1 for metadata. **b**, Pangenome clustered by Jaccard similarity of genome-wide *K*-mers. For the bolded accessions, red denotes hybrid haploid assemblies, green the reference genome and black the chromosome-resolved *Z. marina* and *Z. pacifica* assemblies. **c**, Chromosome 1 (Chr. 1) protein-coding gene synteny. Zmar107-Hum1 (10) was omitted due to its lower-quality assembly. **d**, Hybrid Hap1-*marina* and hybrid Hap2-*pacifica* homologous chromosomes with gene and transposable element density. Transposable elements are grouped as follows: LTR-RTs (long terminal repeat retrotransposons); LINE/SINEs (long/short interspersed nuclear elements); TIRs (terminal inverted repeats). Paired chromosomes are scaled to reflect relative length in Mb.

To test whether *Z. pacifica* could alternatively be described as an ecotype, we compared the *Z. pacifica* genome to both geographically and phenotypically similar *Z. marina*. Leaf width is a plastic trait in *Zostera* and has led species distinctions to be later challenged by genetic evidence, as in the case of the wide-leaved *Z. angustifolia* in Europe^32^. The leaves of *Z. pacifica* are similar to those of higher-latitude *Z. marina*, beginning in Central and Northern California and extending northward along the coastline of North America. We collected *Z. marina* from across six distinct populations in California that display a wide-leaf phenotype and produced un-scaffolded de novo genome assemblies to produce a small pangenome (Fig. 2a,b)^16^. All *Z. pacifica* assemblies cluster distinctly from *Z. marina* across California, with no grouping of *Z. pacifica* with *Z. marina* by either geographical proximity (*Z. pacifica* with Southern California *Z. marina*) or the wide-leaf phenotype (*Z. pacifica* with Northern California *Z. marina*). This supports *Z. pacifica* speciation, as opposed to its being an ecotype displaying wider leaves in response to the darker and colder offshore environment.

Across the *Z. marina* accessions, we did note a persistent chromosome 1 inversion (Fig. 2c). While chromosomes 2–6 are syntenic across the pangenome, a chromosome 1 inversion appears in Southern and Central California accessions Zmar913-Mar3, hybrid Hap1-*marina* and Zmar1017-Elk1. *Z. pacifica* does not share this inversion, so we assume that *Z. pacifica* divergence predates it and that the inversion is not relevant to the speciation. However, this inversion does result in the hybrid possessing an internal inversion between otherwise homologous autosomes.

The *Z. pacifica* genome, assembled and scaffolded into chromosomes as Zpac1022 and hybrid Hap2-*pacifica*, is moderately smaller than that of *Z. marina*, which has more long terminal repeat retrotransposons in the centromere region (Fig. 2d). Centromere assembly in *Zostera* is highly variable across sequencing methods and plant samples, but the smaller genome size and reduced long-terminal-repeat-retrotransposon content of *Z. pacifica* remains consistent between Hap2-*pacifica* and Zpac1022 as compared with their *Z. marina* counterparts (Supplementary Table 1). While one un-scaffolded *Z. pacifica* assembly is an outlier (Zpac1022-Sio1), when we aligned the contigs to a scaffolded assembly, we found that the alignment matched the expected *Z. pacifica* genome size. Querying orthogroups constructed from 29 flowering plants (Supplementary Table 2), we found very few orthogroups with significant *Z. pacifica* gene expansion, and of these new genes that have informative annotations with Gene Ontology (GO) descriptions, nearly all appear to be untranscribed pseudogenes (Supplementary Table 3). This suggests that *Z. pacifica* divergence from *Z. marina* is not the result of conspicuous copy number expansion. Instead, evolving gene function and transcriptional regulation may drive ongoing adaptation, necessitating an experimental approach.

### *Z. marina* and hybrid transcriptomes diverge under low-light conditions

**Fig. 3:**
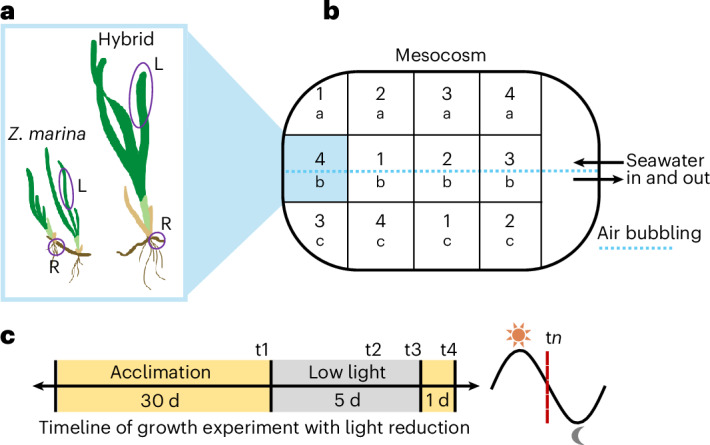
Experimental mesocosm design for low-light challenge. **a**–**c**, The mesocosm, maintained outdoors with flow-through seawater, contained transplanted hybrid and *Z. marina* plants growing in a common garden. At the end of each sampling day before, during and after dark treatment, plants were removed, and leaf (L) and rhizome (R) tissues were collected for nucleotide extraction (**a**). The tank was fed with continuously flowing seawater at ambient ocean temperature, and air was bubbled in along the central axis. Experimental time points 1–4 and replicates a–c are shown (**b**). The experimental timeline contained an acclimation period, a light sample (t1) and two low-light samples at three days (t2) and five days (t3), after which the tank was uncovered, and a recovery time point (t4) was taken after a day. All tissue samples were collected just prior to sunset (**c**).

We next explored whether the hybrid and its *Z. pacifica* genes demonstrate a low-light response distinct from that of *Z. marina*. We conducted a mesocosm growth experiment that challenged the hybrid and *Z. marina* to five days of shading, and we surveyed the transcriptome before, during and after perturbation (Fig. 3a–c). This light reduction was designed to mimic an oceanic storm or plankton bloom that creates semi-darkness at the benthos, persisting for days to weeks. At peak midday sunlight, the light-limited tank received ~37 μmol m^−2^ s^−1^ PAR; previous experimental studies in *Zostera* have simulated low-light stress using between 20 and 52.8 μmol m^−2^ s^−1^, yielding integrated daily light availability <1 mol m^−2^ d^−1^, which constitutes severe shading in the field^8,13,14,33^. Specifically, 3 mol m^−2^ d^−1^ has been defined as the threshold for sustained *Z. marina* growth^21^. Late-day sampling minimized ambient light intrusion and captured the plants’ transcriptomic response to either satiation or starvation of photosynthate.

**Fig. 4:**
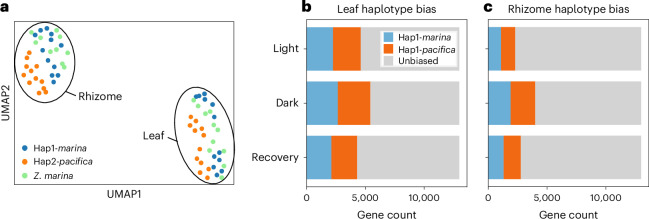
Expression biased to haplotype in the hybrid. **a**, In resolving the Hap1-*marina* and Hap2-*pacifica* transcriptomes, we observed that they occupy distinct space in a Uniform Manifold Approximation and Projection (UMAP) dimensionality reduction of all experimental time points. The UMAP represents the expression of 1:1 orthologous gene pairs across all tissues and treatments. Hybrid Hap1-*marina* clusters with inbred *Z. marina* in both leaves and rhizomes. **b**,**c**, We extracted the genes differentially expressed between the hybrid haplotype 1:1 orthologues at each time point to derive gene counts for biased expression. In the leaf (**b**) and rhizome tissue (**c**), a subset of genes display expression bias to Hap1 or Hap2. The quantity of biased gene pairs varies over experimental time points and between tissues. Supplementary Data 3 presents the 1:1 orthologue pairs.

**Extended Data Fig. 6:**
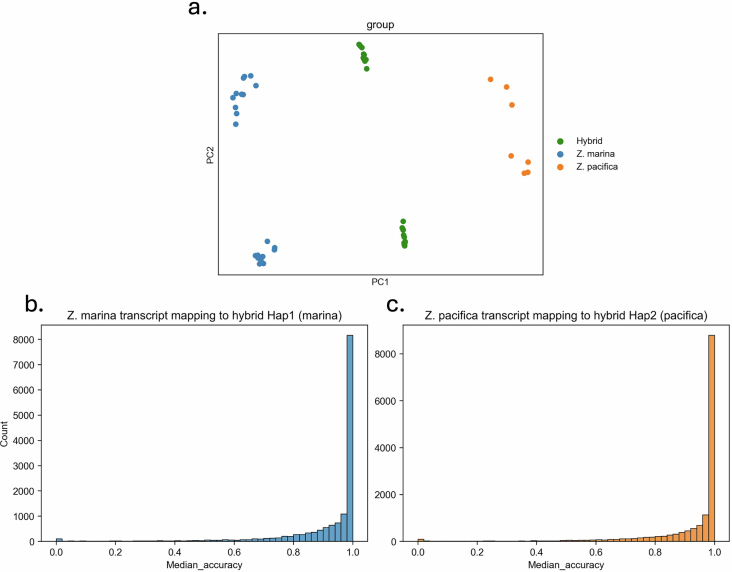
RNAseq mapping to hybrid reference transcriptome identifies accurately mapping 1:1 orthologs for confident differential expression analysis. There are 16,336 1:1 gene orthologs between the hybrid haplotypes after excluding ambiguous pairings of one to many. Within this ortholog set, we established transcript mapping accuracy using a dataset of inbred *Z. marina* and *Z. pacifica* RNA-seq. **(a)** All tank experiment *Z. marina* and Hybrid, as well as wild-collected *Z. pacifica* from Scripps Institution of Oceanography, clustered in PCA space when mapped to the Hybrid transcriptome. The *Z. marina* and *Z. pacifica* transcripts were used to ascertain mapping accuracy of reads to the Hybrid haplotypes, with **(b)** *Z. marina* mapping to Hap1 with >90% accuracy for 73.5% of 1:1 ortholog genes and **(c)** *Z. pacifica* mapping to Hap2 with >90% accuracy for 76.5% of 1:1 ortholog genes. 85.4% of Hybrid reads map to these >90% confident gene pairs, over both haplotypes.

**Extended Data Fig. 7:**
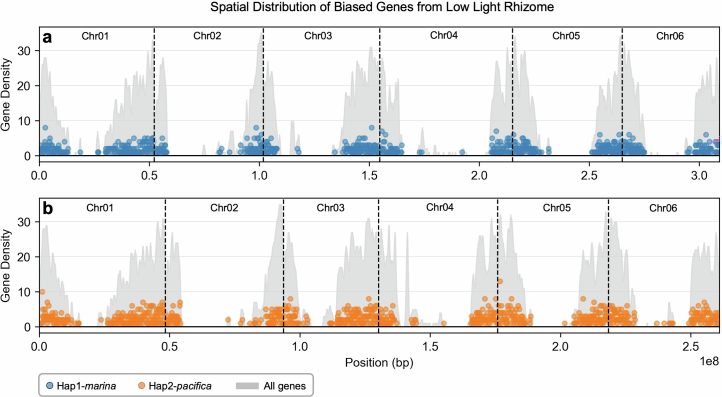
Distribution of biased genes in the Hybrid genome. Haplotype expression bias is consistent with gene density and does not saturate a specific chromosome or region on either haplotype. We visualize 1:1 gene orthologs biased to **(a)** Hap1-*marina* and **(b)** Hap2-*pacifica* in the low light rhizome, as an example of this. Gene density is defined as gene count within a 150 kb window.

The mesocosm experiment presents a combination of hybrid and inbred (non-hybrid) *Z. marina* RNA-seq samples; we mapped all RNA-seq reads to the hybrid transcriptome, resulting in an average of 69.8% mapping for the hybrid samples and 70.2% for *Z. marina* (Supplementary Table 4). To eliminate haplotype cross-mapping prior to differential expression analysis, we used inbred *Z. marina* and *Z. pacifica* RNA-seq to calculate transcript mapping accuracy for every Hap1–Hap2 orthologous gene pair (Extended Data Fig. 6a–c). After applying a 90% mapping accuracy cut-off, we carried through 13,952 1:1 orthologues to differential expression, representing 85.4% of the transcription observed in the hybrid throughout the study as a function of transcripts per million (Supplementary Data 3). With this set of orthologues, we observed that transcriptome-wide patterns of transcript relative abundance in the hybrid Hap1-*marina* haplotype are similar to those in inbred *Z. marina* (Fig. 4a). A subset of genes in the hybrid demonstrate expression bias between the parental haplotypes (Fig. 4b,c). Across all conditions, bias is higher in the leaf than in the rhizome but does not favour one subgenome. Biased expression is distributed evenly across the genome, suggesting genome-wide divergence of regulatory timing or strength within gene networks, rather than a single region experiencing selective pressure (Extended Data Fig. 7a,b).

**Fig. 5:**
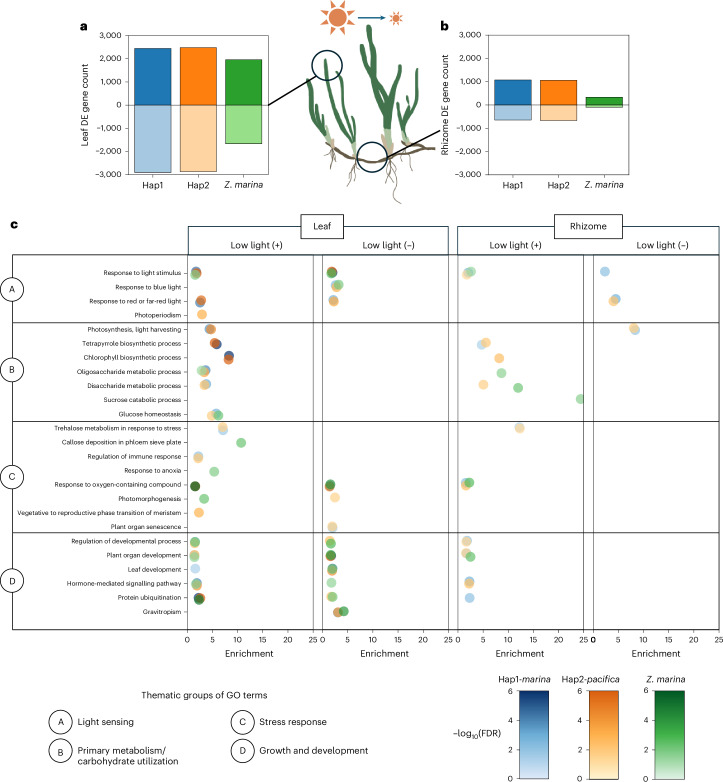
Differential expression in the hybrid and *Z. marina* exposed to low light. **a**,**b**, Counts of differentially expressed (DE) genes in the leaf (**a**) and rhizome (**b**) for hybrid Hap1-*marina*, Hap2-*pacifica* and *Z. marina*, with positive numbers indicating upregulation in the shade, and negative numbers indicating downregulation. Shaded time points t2 and t3 in combination were compared to full-light time points t1 and t4 to extract DE genes. **c**, GO category enrichment analysis of all up-regulated (+) and downregulated (−) DE genes, with all points plotted exceeding a significance cut-off of FDR < 0.05. GO terms are presented with descriptive names and grouped into thematic groups. Exhaustive results of the GO enrichment analysis, including GO ID numbers, are presented in Supplementary Data 5.

Within the set of 13,952 confident orthologue pairs, we carried out differential expression analysis of the plants in low light (t2 and t3) compared with full light (t1 and t4) (Supplementary Data 4) and identified enriched GO terms that describe divergent trends for the hybrid and inbred *Z. marina*. By separating the hybrid haplotypes in this analysis, we could directly compare the Hap1-*marina* and Hap2-*pacifica* subgenomes with *Z. marina* in terms of gene count. A potential weakness of this approach is that *Z. marina* as a diploid may demonstrate an amplified differential expression signal when orthologous genes on both haplotypes are summed, but we did not observe this and in fact found fewer differentially expressed genes in *Z. marina*. Of the two tissue types, the leaf experienced greater up- and downregulation of genes in response to low light intensity, with the hybrid leaf upregulating 2,445 genes on Hap1 and 2,481 on Hap2, and the *Z. marina* leaf upregulating 1,959, whereas the hybrid rhizome upregulated 1,073 and 1,062 genes and the *Z. marina* rhizome 334 (Fig. 5a,b).

We extracted enriched GO groupings from the up- and downregulated gene sets (Fig. 5c and Supplementary Data 5). Light-sensing genes (A) experience up- and downregulation for both *Z. marina* and the hybrid, though the hybrid’s response to red or far-red light and photoperiodism is significant compared with that of *Z. marina*. Blue light sensing is downregulated in both plants. A divergent trend emerges in primary metabolism and carbohydrate utilization (B), as the plants respond to low photosynthate. At this late-day collection, the hybrid has upregulated photosynthesis (enrichment, 4.4 (Hap1) and 4.7 (Hap2); false discovery rate (FDR), <0.0001). Additionally, genes for chlorophyll biosynthesis are highly enriched in the hybrid leaf (enrichment, 8.2; FDR < 0.0001) and not in *Z. marina*. Instead, we observed *Z. marina* upregulating sucrose catabolism in the rhizome (enrichment, 24.3; FDR < 0.05). The hybrid also upregulates sucrose catabolism, but enrichment is statistically significant only in *Z. marina* due to lower differentially expressed gene counts. From this, we determined that both *Z. marina* and the hybrid catabolize some rhizome sugars during shade stress, but the hybrid additionally maintains photosystem proteins and chlorophylls for photosynthesis, suggesting that photosynthesis continues despite low PAR. For stress response (C), both *Z. marina* and the hybrid up- and downregulate the leaf’s response to radical oxygen species. Otherwise, their stress responses differ, with the hybrid upregulating trehalose metabolism and the regulation of the immune response and reproductive phase shift in the leaf, and the *Z. marina* leaf upregulating callose deposition, response to anoxia and photomorphogenesis. Lastly, growth and development genes (D) demonstrate both up- and downregulation in the hybrid and *Z. marina*, indicating a mix of turning on and turning off pathways due to light reduction.

### Low-light photosynthesis in the hybrid leaf

**Fig. 6:**
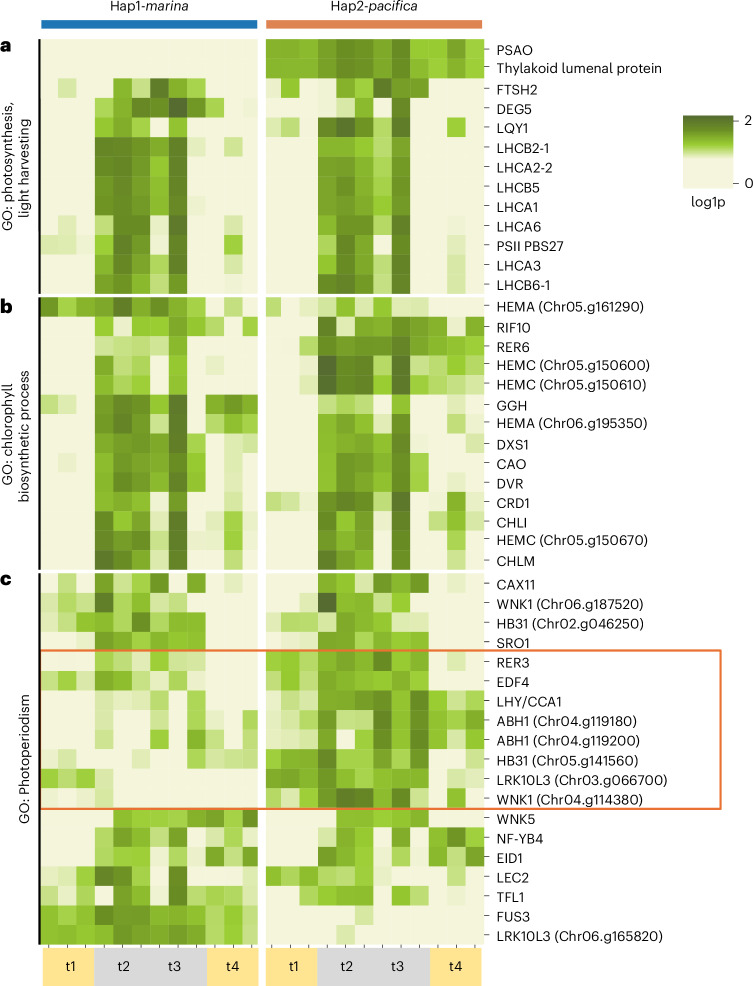
Hybrid leaf upregulation of key GO categories implicates *Z. pacifica* photoperiod regulation. **a**–**c**, Hybrid genes associated with the enriched GO categories of GO:0009765 (photosynthesis, light harvesting) (**a**), GO:0015995 (chlorophyll biosynthetic process) (**b**) and GO:0009648 (photoperiodism) (**c**), visualized with normalized log fold change across the experimental time points for the hybrid leaf experiencing low-light stress. The orange box highlights Hap2-*pacifica* biased overexpression in the photoperiodism group. Gene identity was derived from gene annotations and *A. thaliana*/*O. sativa* orthologues. Experimental replicates are clustered on the horizontal axis by transcriptome (Hap1-*marina* and Hap2-*pacifica*) and time point (t1 (full light), t2 and t3 (low light), and t4 (full-light recovery)). Gene positional information (for example, chr04.g119180) is given for the Hap1-*marina* orthologue in cases where multiple gene copies are expressed. The log1p values plotted represent normalized expression values, log-transformed for visualization.

**Extended Data Fig. 8:**
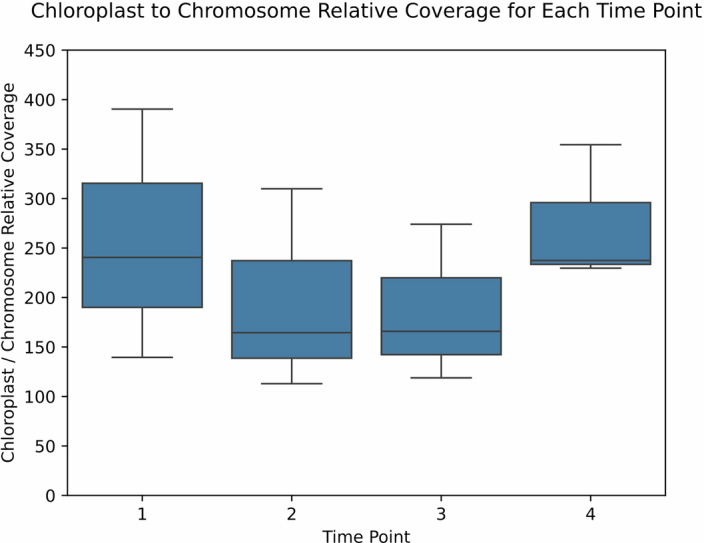
Chloroplast counts derived from read coverage. Illumina DNA sequencing of the Hybrid leaf at each timepoint yielded read coverage of the nuclear genome and chloroplast genome. Relative coverage is the ratio of chloroplast-aligning reads to nuclear genome –aligning reads. Each timepoint contains n = 3 replicates, with the center line denoting median, box the interquartile range, and whiskers the minimum and maximum values.

While *Z. marina* responded to the experimental shade conditions by metabolizing its stored rhizome sugars, the hybrid additionally upregulated chlorophyll biosynthesis and late-day photosynthesis, suggesting that a shift in circadian-clock- regulated transcription drives the continued maintenance of photosystems and implied production of photosynthate. Light-harvesting and chlorophyll biosynthesis genes show concerted overexpression across both the Hap1-*marina* and Hap2-*pacifica* orthologues during the low-light time points t2 (three days of shade) and t3 (five days of shade) (Fig. 6a,b). Light-harvesting components including several orthologues of *LIGHT HARVESTING COMPLEX A* and *B* (*LHCA* and *LHCB*), *LOW QUANTUM YIELD OF PHOTOSYSTEM II 1* (*LQY1*) and *PSB27*, encoding a Photosystem II family protein, are highly expressed at t2 and have heterogenous expression at t3 (refs. ^34–36^). Other light-harvesting components—*FTSH2*, encoding an ATP-dependent zinc metalloprotease, and *DEG5*, encoding an ATP-independent serine protease, both involved in PSII maintenance and recycling—show their highest expression at t3 (refs. ^37,38^). Despite strong upregulation of light-harvesting complex transcripts, plastid-to-nuclear read ratios showed no increase in chloroplast number under shade (Extended Data Fig. 8), suggesting that the hybrid copes with low light by upregulating LHCs and photosystem-maintenance genes to repair or replace photosystem components rather than by increasing chloroplast density.

The key light-harvesting pigments chlorophyll *a* and chlorophyll *b* are biosynthesized from glutamate and share a porphyrin scaffold intermediate, protoporphyrin IX, with haem biosynthesis^39,40^. Several key biosynthetic components of chlorophyll biosynthesis are upregulated both upstream and downstream of protoporphyrin IX, indicating concerted upregulation before and after the haem junction. These include the early-pathway HEMA and HEMC, which encode the biosynthesis genes for glutamyl-tRNA reductase (*GluTR*) and porphobilinogen deaminase (*PBGD*), and the down-pathway *CHLI*, encoding a subunit of magnesium-protoporphyrin chelatase, as well as the genes for magnesium protoporphyrin methyltransferase (*CHLM*) and 3,8-divinyl chlorophyllide *a* 8-vinyl reductase (*DVR*)^40^. Additionally, *CAO*, encoding chlorophyllide *a* oxygenase, catalyses the interconversion of chlorophyllide *a* and chlorophyllide *b*, driving chlorophyll *b* biosynthesis^41^. While most components are overexpressed on both haplotypes, one HEMA homologue demonstrates transcription bias to Hap1-*marina*, and HEMC, which shows a tandem duplication on chromosome 5, is biased to Hap2-*pacifica* for two copies.

Though we observed relatively balanced overexpression of photosynthesis and pigment biosynthesis genes across parental 1:1 orthologues, several photoperiod genes demonstrate haplotype bias (Fig. 6c). In plants, photoperiodism describes the ability to respond to day length by modulating time-of-day-regulated functions such as photosynthesis and flowering, and indeed in this GO category we observed several circadian clock components and signalling proteins. The core circadian clock transcription factor gene *LATE ELONGATED HYPOCOTYL* (*LHY*) is upregulated in low light on both haplotypes but is biased to Hap2-*pacifica*^42^. *LHY* transcription oscillates with morning-specific time-of-day-specific expression, when it binds the promoters of light-harvesting complex encoding genes to promote transcription and phytochrome signalling^42,43^. *LHY* transcript oscillation has been observed in *Z. marina* to peak in the pre-dawn under ideal light conditions, but we measured transcript upregulation in the late-day hybrid subjected to low light^12^. Abiotic cues reach the circadian clock through signalling pathways; ABA signalling has previously been implicated in the *Zostera* low-light stress response, and we note two copies of an mRNA binding protein encoding gene, *ABH1*, involved in post-transcriptional regulation of ABA signalling with overexpression strongly biased to Hap2-*pacifica*^13,44^. WITH NO-LYSINE KINASEs (WNKs) are protein kinases implicated in integrating abiotic stress signals into the timing of the circadian clock, evidenced in monocots such as rice (*Oryza sativa*) and soy (*Glycine max*)^45–47^. Three WNKs are overexpressed in the low-light hybrid, including a chromosome 4 *WNK1* homologue unique to temperate seagrasses that only shows low-light differential expression of the Hap2-*pacifica* orthologue, indicative of neofunctionalization. The WNKs are also involved with ABA signalling, but these interactions are not fully elucidated^46^. *WNK1* may also act on flowering time genes (*FT*s) such as *TIME OF FLOWERING 1* (*TFL1*), which we observed to be upregulated in this GO category with heterogeneity among replicates^47^. Lesser-studied genes implicated in GO:photoperiodism include the zinc-finger homodomain transcription factor *HB31*, involved in floral phenotype, and the serine/threonine protein kinase *LRK10L3*, each of which has multiple copies in the hybrid, demonstrating haplotype bias^48,49^.

**Fig. 7:**
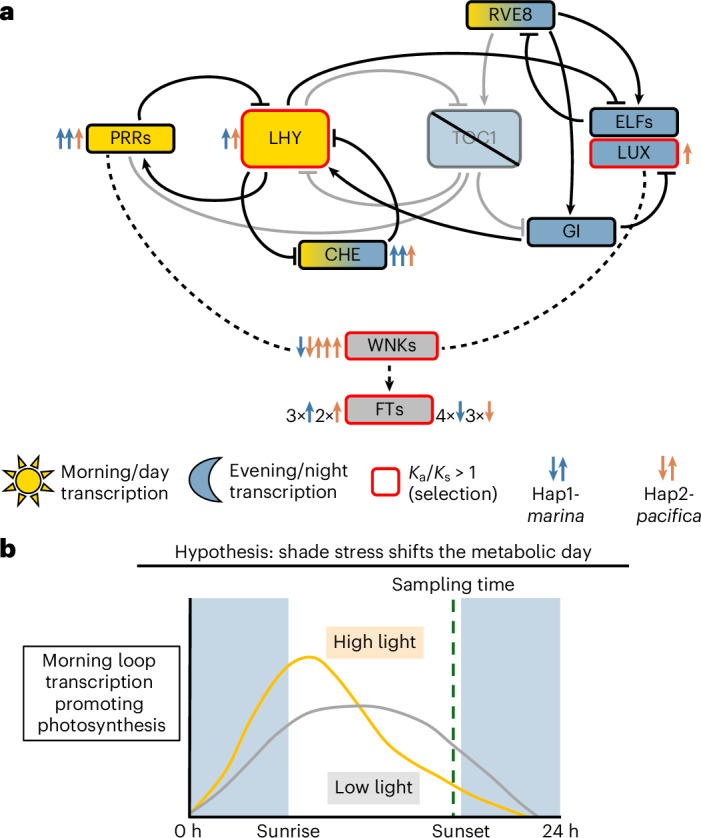
The circadian clock morning loop is overexpressed in the late day under shade conditions. **a**, Using gene homology to *Arabidopsis*, we extracted the clock genes embedded in cycles of activation and repression, with the morning loop promoting photosynthesis and the evening loop promoting respiration. The regulatory relationships of circadian clock components are represented in a network with connecting lines ending in arrows indicating activation and those ending in T’s indicating repression. The up and down arrows indicate differential expression, with each arrow indicating a gene copy and its direction corresponding to up- or downregulation. The loss of TOC1 in *Zostera* is represented as a greying out of those regulatory connections. Gene information, including *K*_a_/*K*_s_ values and log fold changes, are presented in Supplementary Table 5. **b**, Observations made in this late-afternoon sampling procedure support a theory that adaptive *Zostera* circadian clock regulation shifts periodicity under low-light conditions.

**Extended Data Fig. 9:**
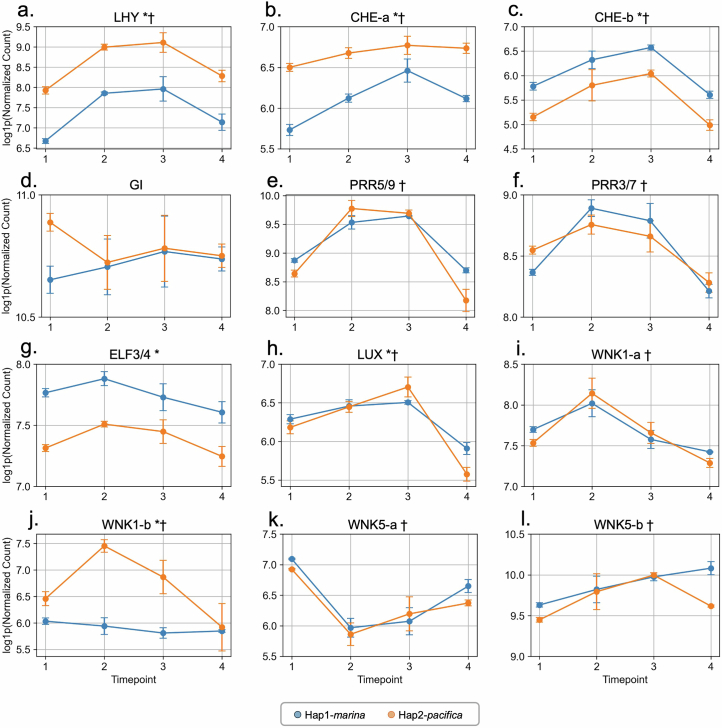
Hybrid clock genes. **(a-l)** Circadian clock gene expression across experimental timepoints in the Hybrid. Genes with (*) demonstrate haplotype biased expression and those with (†) differential expression of shade timepoints [2,3] compared to timepoint [1]. Verbose gene descriptions are present in Supplementary Table 5. Each data point represents the average of n = 3 replicates, and the error bar gives standard deviation.

In addition to LHY, we observed other circadian clock transcription factors to be upregulated in low light, particularly those comprising the morning loop that promotes photosynthesis, identified using homology to *Arabidopsis thaliana* and *O. sativa* (Fig. 7a and Supplementary Table 4)^50^. Several of these genes show haplotype bias (Extended Data Fig. 9a–l). The *PSEUDO-RESPONSE REGULATOR*s (*PRR*s) are activated by *LHY* and expressed in the morning and midday, and they have three orthologues in *Zostera*, two of which are expressed and are upregulated across both haplotypes of the hybrid^42,43^. The classic repressor of *LHY* and core constituent of the evening loop, *TIMING OF CAB EXPRESSION 1 / PSEUDO-RESPONSE REGULATOR 1* (*TOC1/PRR1*), is highly conserved in land plants but not present in *Zostera*^16^. In the evening complex, *LUX ARRHYHTHMO* (*LUX*) is upregulated, but the differential expression of *EARLY FLOWERING* *3* or *4* (*ELF3*/*4*) is not significant. Along with *LHY*, the *ELF3/4* and *CCA1 HIKING EXPEDITION* (*CHE*) genes demonstrate haplotype bias across all time points, with *LHY* and one *CHE* biased to Hap2-*pacifica*, and the other *CHE* and *ELF* biased to Hap1-*marina*.

The majority of these circadian genes are experiencing purifying selection, with the ratio of protein sequence synonymous to non-synonymous substitution rates (*K*_a_/*K*_s_) <1 between hybrid Hap1-*marina* and Hap2-*pacifica* orthologues. However, the upregulated *LHY*, *LUX*, *WNK5* and *TFL1* are experiencing positive selection, with *K*_a_/*K*_s_ values of 1.05, 1.26, 1.22 and 1.16, respectively (Supplementary Table 5). Our RNA-seq collections represent a single time point on the oscillator. Our results therefore support a theory of shifting day length that will require testing in a time course in the future (Fig. 7b).

## Discussion

Through whole-genome sequencing (WGS) and assembly of resolved parental chromosomes, we have described the landscape of a *Zostera* hybrid genome, which is a first-generation F_1_ homoploid with *Z. marina* and *Z. pacifica* parental haplotypes that have yet to recombine. The haplotype-resolved hybrid transcriptome enabled the comparison of *Z. marina* and *Z. pacifica* gene orthologues under shade conditions. The hybrid demonstrated a divergent low-light response from inbred *Z. marina*, upregulating photosystem and chlorophyll biosynthesis; this differential expression implies the continued creation of photosynthate at a light intensity above the light compensation point, or the minimum light intensity for carbon assimilation, indicative of shade tolerance (though direct measurements of rhizome sucrose are absent from this study).

The observation of late-day photosynthesis gene upregulation in the hybrid may be related to photoperiod control, as we observed differential expression of circadian clock transcription factors, *WNK* protein kinases and ABA signalling proteins. Under shade conditions, we observed the morning loop of the circadian oscillator to be upregulated in the evening. Light reduction is known to lengthen the circadian period—for example, the phase of *LHY* is advanced by two hours in shaded tomato, and hybrid haplotypes have been shown decouple the oscillation of core clock genes^51,52^. *LHY* demonstrated expression bias to Hap2-*pacifica* over the course of our experiment, suggesting that the *Z. pacifica* and *Z. marina* clocks may have asynchronous daily oscillations.

*Zostera* species possess a single *LHY* homologue per haplotype, while some plant lineages (including the Brassicaceae, with the model plant *Arabidopsis*) have two, performing semi-redundant tasks^16,42^. Ma et al. hypothesized that the reduction of clock genes in *Zostera* and other aquatic plants is due to water availability reducing the need for such strict timing of metabolic tasks^16^. Building on this, we posit that shade tolerance in temperate seagrasses, exemplified by the deep-living *Z. pacifica*, favours a reduced clock that is flexible to shifting periodicity. These plants face a highly variable light environment; storms or plankton blooms may transiently drop PAR to near the light compensation point for days to weeks, while seasonal shifts set longer-term constraints on growth, necessitating the retention of clock-controlled processes such as flowering. This lifestyle favours a plant with a malleable definition of day length to take advantage of all photosynthetically available light during short-timescale low-light events, while retaining clock-regulated transcription given ideal light conditions. The circadian clock in *Zostera* deserves deeper study directed towards potential neofunctionalization of these conserved clock genes across the metabolic day as important players in low-light resilience.

The *Z. marina* × *pacifica* hybridization event in Mariner’s Basin was a consequence of mitigation management. Yet, this population may be useful in restoration if advantageous traits such as low-light tolerance or high growth rate from heterosis are demonstrated in the field. Ecological studies in the mixed meadow could compare primary productivity and bed expansion/contraction across multiple seasons to determine whether the hybrid is becoming a competitive dominant at the site. Longer-term in situ shading experiments or experimental small-scale outplanting of hybrid and *Z. marina* plants in a light-challenged site, coupled with continuous PAR measurements, could explore survivorship and growth rate. Additionally, the vital question of sexual viability remains. The dominant hybrid clone appears to be first-generation, or F_1_, which aligns with the recency of the *Z. pacifica* transplant at the site. Though we have yet to observe clear F_2_ progeny through exploratory genotyping, the hybrid can produce flowers. After no observed flowering in 2023–2024, in summer 2025, the hybrid produced reproductive tissue, flowers and seeds, indicating the potential for outcrossing. Eelgrass can produce extensive clones, though these are less common in Southern California than genotypically mixed beds that rely on some amount of sexual reproduction^18^. It remains unclear whether the prevalence of the clone is due to low sexual viability or to having a competitive genotype suited to its environment. Discovering whether the hybrid produces viable seeds, and the frequency of these flowering events moving forward, is a priority for understanding its ecological impact. Dependent on these traits, the hybrid could expand the range of suitable habitat for eelgrass restoration, allowing outplanting in areas impacted by lower PAR from depth, seasonal events or poor water clarity.

Light limitation will continue to hamper *Z. marina* restoration efforts as eutrophication, dredging and sea level rise contribute to reduced PAR for bay and estuarine eelgrass. Expanding eelgrass restoration to include offshore *Z. pacifica* could benefit coastal ecosystems and carbon capture in Southern California but requires further elucidation of *Z. pacifica* growth parameters for selecting suitable restoration sites, which the hybrid may enable^34^. *Z. marina* bay and estuarine systems and *Z. pacifica* open coast ecosystems are differentiated by a host of biophysical factors, even as their genomes appear highly similar in gene content and synteny. For comparative studies, the hybrid offers a clonal accession containing both genomes that is suitable for ex situ growth experiments. We encourage future studies of the hybrid directed towards these experimental applications, as well as ecological surveys of hybrid growth rates, reproduction and low-light tolerance in the field.

## Methods

### Plant collection for nucleotide extraction

For all plants subjected to WGS, complete sample metadata (locations throughout California and dates of collection) are presented in Supplementary Table 1. All *Z. marina* for WGS were collected by snorkelling from these various locations, while *Z. pacifica* for WGS and RNA-seq was collected in the Matlahuayl SMR in San Diego, California, USA, via SCUBA. Using gloved hands, whole seagrass ramets (root, rhizome and leaves) were carefully cut from the clonal rhizome. On shore or boat, each specimen was briefly cleaned in seawater of epiphytes and sediment, separated into root, rhizome and leaf tissue samples, and flash-frozen in liquid nitrogen. For high-molecular-weight (HMW) DNA, we used the leaf tissue within the sheath, down to the first node of the rhizome. *Zostera* plants were collected and sacrificed or maintained in seawater tanks under California Department of Fish and Wildlife scientific collection permit no. S-210200011-21023-001.

### Plant collection for transplant

In September 2023, 12 hybrid and 12 *Z. marina* were collected from Mission Bay at the same depth (2–3 m), bagged and transported in seawater in a chilled cooler. The transplants all contained two to four ramets and appeared healthy (that is, substantial new leaf growth, young roots and minimal epiphytes). In parallel with the plant collection, we collected several pounds of sediment from the eelgrass beds to recreate the rhizosphere and sediment microbiome in the eelgrass beds to aid plant acclimation and mixed this with more accessible sediment from the shoreline to increase the sediment depth in the tank to ~10 cm. A turquoise outdoor aquaculture tub tank (base dimensions 0.5 m by 1.1 m) at Scripps Institution of Oceanography was outfitted with flowing seawater at ambient ocean temperature and air bubblers down the centre of its long axis and shaded by a mesh cover to reduce tank overheating and protect the tank from birds. Hybrid and *Z. marina* plants were planted in a common garden in the tank. Figure 3 provides a schematic of the tank and the experimental design. The plants were acclimated for 30 days before exposure to experimental conditions. During this acclimation period, the tank and plants were cleaned of algal epiphytes and grazers, and mature leaves were trimmed to remove necrotic tissue. *Phyllaplysia* slugs were allowed to remain in the tank to reduce algal epiphytes. Transplant was deemed successful when we observed new leaf growth of existing ramets, as well as several juvenile ramets appearing, around 20 days after transplant for both genotypes. A prior validation of these growth conditions was conducted with a non-experimental seagrass tank that supported both hybrid and *Z. marina* eelgrass for several months.

### Growth experiment

We constructed a shade box from heavy-duty white cardboard on a wood frame to entirely surround the tank. At peak sunlight, the box reduced available light to the plants to an average of 37 μmol m^−2^ s^−1^ (~95% reduction) across the experiment days, measured at noon each day using an Odyssey Xtreem PAR logger (OXLPAR), calibrated by an Apogee MQ-510 quantum meter. Due to an error with the PAR logger, PAR measurements at other times of day were not collected; we therefore infer a daily integrated PAR of <1 mol m^−2^ d^−1^ not from direct measurement and integration but from comparison to other studies in *Zostera* spp.^13,14,21^. The water temperature in the tank averaged 17.9 °C and reached a minimum of 16.7 °C on the fourth day of the experiment, which coincided with a swell bringing colder sea surface temperatures. The experiment consisted of four collection time points, all carried out at sunset: t1 before shade treatment (control), t2 at day 3 of shade treatment, t3 at day 5 of shade treatment and t4 the day after the box was removed. Each sampling took three plants of each genotype as biological replicates, and the youngest ramet was sampled for leaf and rhizome tissue (excluding juvenile ramets <10 cm tall). To normalize by growth stage across all samples, leaf lengths as a proxy for age were kept relatively consistent, with hybrid leaves 22.1 cm ± 5.7 cm and *Z. marina* 15.8 cm ± 5.8 cm. Rhizomes were sampled at the first internode of the same ramet. To reduce sampling bias of microenvironments within the tank at any given time point (for example, closest to the seawater outflow or next to the tank wall), replicates a–c for each time point were distributed throughout the tank. All leaves were cleaned of epiphytes (minimal), roots and sediment were removed from the rhizomes, and the samples were flash-frozen in liquid nitrogen and transported on dry ice. Each plant was processed separately, and the box was closed between each sampling.

### DNA extraction

For the hybrid (Zmar912-Mar1), Zpac1022-Sio1, Zmar1010-Tom3, Zmar1017-Elk1, Zmar108-Cre1, Zmar106-Eel1 and Zmar1015-Sfb1, HMW DNA was extracted using an Oxford Nanopore Community protocol for HMW DNA, which uses components of the QIAGEN Blood and Cell Culture DNA Midi Kit (13343) (https://community.nanoporetech.com/extraction_method_groups/plant-leaf-gDNA). We followed this protocol with two modifications: a 20-ml lysis buffer ‘half reaction’ to eliminate sample splitting, and a three-hour final isopropanol incubation.

For Zmar913-Mar3, Zpac1022-Sio2 and Zmar107-Hum1, HMW DNA was extracted using the Circulomics Nanobind Plant Nuclei Big DNA Kit (NB-900-801-01), following the UHMW DNA Extraction protocol from isolated plant nuclei (EXT-PLH-002). To isolate nuclei, we used the following procedure. After 10 min of grinding in liquid nitrogen, ~1.5 g of leaf powder was resuspended in IBTB buffer (15 mM Tris-HCl, 10 mM EDTA, 130 mM KCL, 20 mM NaCL, 8% PVP-10, 1.15 mM spermine, 1.15 mM sperimidine, 0.1% Triton-X 100, 7.5% 2-mercaptoethanol (BME)). Before the spermine and spermidine were added, the pH was adjusted to 9.5, and the Triton-X and BME were added immediately before use. The following isolation steps were performed on ice: 100-μm and 40-μm cell strainers filtered out cell debris and organelles lysed by adding 0.25% Triton-X 100. Residual cell debris were removed via 1 min of centrifugation at 60 *g* before the nuclei were pelleted at 2,500 *g* for 10 min, followed by washes in IBTB. Nuclei containing traces of green plastid material were then carried into the Circulomics protocol with no alterations. For seagrass HMW samples, we recommend the Nanopore protocol, as used for the hybrid, which carries out direct lysis and extraction on young plant tissue; the nuclei isolation method represents an older workflow.

DNA intended for short read sequencing was extracted using a Qiagen DNeasy Plant Mini Kit (96106). An MP Biomedicals FastPrep-96 bead beater (6010500) was used for tissue homogenization; <100 mg of all samples were placed in MP Biomedicals 2-ml steel bead beating tubes (6925050) on dry ice and beaten for 90 seconds at maximum speed with dry ice before we proceeded to the lysis step.

All DNA was quality assessed using a ThermoFisher Nanodrop 2000c (8353-30-0011) and a Qubit Fluorometer (Q33238) with the Broad Range assay (Q33231). Fragment lengths of all HMW DNA samples were measured using an Agilent 4150 Tapestation (G2992AA) with a Genomic DNA ScreenTape (5067-5365).

### RNA extraction

The mesocosm experiment yielded leaf and rhizome tissue for RNA extraction, and several wild-collected *Z. pacifica* tissue samples were included to aid transcript identification. We present RNA integrity and yields for all tank experiment samples in Supplementary Table 3. An MP Biomedicals FastPrep-96 bead beater (6010500) was used for tissue homogenization; <100 mg of all samples were placed in MP Biomedicals 2-ml steel bead beating tubes (6925050) on dry ice and beaten for 90 seconds at maximum speed with dry ice before we proceeded to lysis. We used a Qiagen RNeasy Plant Mini Kit (74904) with no modifications. All RNA samples were treated with ThermoFisher Invitrogen TURBO DNase and DNase inactivation reagent according to the manufacturer’s instructions (AM1907). Rhizome samples from *Z. marina* time points t3 and t4 yielded negligible RNA, despite several repeated extractions, leading to loss of a replicate at t3 and t4 for *Z. marina* rhizome, and one replicate lost at t4 for *Z. marina* leaf (Supplementary Table 4). RNA samples were quantified and quality assessed using an Agilent 4150 Tapestation (G2992AA) with RNA ScreenTape (5067-5576).

### Long read library preparation and sequencing

Coverage statistics of sequencing reads from Oxford Nanopore Technologies (ONT) and Pacific Biosciences (PacBio) for individual assemblies are presented in Supplementary Table 1. For Zmar913-Mar3, Zpac1022-Sio2 and Zmar107-Hum1, samples for ONT were prepped for sequencing via ligation (SQK-LSK110) using the Long Fragment Buffer included in the kit, and immediately loaded onto a PromethION flow cell (v.9.4.1) and run on the PromethION running Guppy (v.5.0.12) for base calling and visualization with MinKNOW (v.21.05.20). For Zmar913-Mar3, a 250-ng library prep yielded 13,270,000 reads, 62.59 Gb passed bases and a sequencing N50 of 21.89 kb. A 266-ng Zpac1022-Sio2 library prep yielded 14,940,000 reads, 94.12 Gb passed bases and a sequencing N50 of 16.21 kb.

The hybrid Zmar912-Mar1 was sequenced on a PacBio Sequel II, with SMRTbell 3.0 library prep (102-141-700). A single SMRT cell yielded 1,550,000 HiFi reads (QV > Q20) with a mean length of 16.54 kb, resulting in a 25.58-Gb yield with a mean QV of Q31. Zpac1022-Sio1, Zmar1010-Tom3, Zmar1017-Elk1, Zmar108-Cre1, Zmar106-Eel1 and Zmar1015-Sfb1 were sequenced on a PacBio Revio with SMRTbell library prep for Revio (103-381-200) and barcoded in groups of two or three per SMRT cell, with the individual SMRT cells producing 86.40–93.70 Gb each.

### Short read library preparation and sequencing

WGS libraries for the hybrid clonal genotyping sample set as well as for the hybrid genome assembly were prepared using an Illumina Nextera Flex (20018706). The WSG libraries for samples Zmar913-Mar3 and Zpac1022-Sio2 were prepared via ThermoFisher Collibri ES DNA library prep for Illumina (A38605024). The cDNA library preps of all RNA-seq samples were prepared via Illumina Stranded mRNA library prep (20040534), which includes a Poly-A selection. For Zmar913-Mar3, Zpac1022-Sio2 and Zmar912-Mar1, high-throughput chromatin conformation capture (Hi-C) libraries were prepared with a Phase Genomics Proximo Hi-C kit for plant tissue (KT3045) with protocol v.4.0. All library preps were completed according to the manufacturer’s instructions, with no protocol modifications. The hybrid Zmar912-Mar1 required a repeat library prep and sequencing to achieve quality scaffolding, and the Hi-C coverage in Supplementary Table 1 reflects a summed yield. Hi-C and non-hybrid WGS libraries were sequenced on an Illumina NextSeq500, and cDNA libraries and hybrid WGS libraries on a NovaseqX, all with the PE150 run configuration. Coverage statistics of sequencing reads from Illumina WGS libraries and Hi-C libraries for individual assemblies are presented in Supplementary Table 1. RNA-seq mapping rates and yields from each Illumina cDNA library are presented in Supplementary Table 3.

### Genome assembly

PacBio HiFi, ONT and Illumina HiC reads were incorporated into the assembly of the Zmar912-Mar1 hybrid with HiFiasm v.0.19.8 (https://github.com/chhylp123/hifiasm)^53^. ONT reads longer than 30 kb and with a mean quality greater than Q10 were used as the ‘ul’ input into HiFiasm, while ONT reads shorter than 30 kb with a mean quality greater than Q20 were combined with the PacBio reads as HiFi input. ONT read filtering was accomplished using fastq-filter v.0.3.0. The HiC reads were included via the ‘hic1’ and ‘hic2’ flags to improve assembly phasing. After assembly, the contigs were screened for contamination using v.0.5.0 of NCBI’s Foreign Contamination Screening–GX workflow (https://github.com/ncbi/fcs)^54^. PacBio HiFi reads from Zpac1022-Sio1, Zmar1010-Tom3, Zmar1017-Elk1, Zmar108-Cre1, Zmar106-Eel1 and Zmar1015-Sfb1 were assembled and filtered with the same versions of HiFiasm and Foreign Contamination Screening–GX as the hybrid.

Prior to assembly, the longest ONT reads from Zmar913-Mar3 and Zpac1022-Sio2 were sub-sampled to produce 13.5 Gb of data per sample, reducing read coverage to around 60×. ONT reads from the Zmar913-Mar3, Zpac1022-Sio2 and Zmar107-Hum1 samples were then assembled with Flye v.2.9 (https://github.com/mikolmogorov/Flye)^55^. The contigs for these samples were then polished with three rounds of Racon v.1.4.20 (https://github.com/isovic/racon)^56^. An additional three rounds of Pilon v.1.24 (https://github.com/broadinstitute/pilon) polishing were then used for Zmar913-Mar3 and Zpac1022-Sio2 with short-read data^57^.

### Genome scaffolding and resolving chromosomes in the hybrid

Illumina HiC reads were mapped to both haplotypes of the Zmar912-Mar1 hybrid using bwa (https://github.com/lh3/bwa) v.0.7.17-r1188 and samblaster v.0.1.26 (https://github.com/GregoryFaust/samblaster)^58,59^. Filtering of the mapped reads, followed by ordering and orientation of the contigs, was accomplished with the HapHiC v.1.0.3 pipeline (https://github.com/zengxiaofei/HapHiC)^60^. HiC reads from the Zmar913-Mar3 and Zpac1022-Sio2 samples were mapped to their respective contigs using the Juicer v.1.6.2 pipeline (https://github.com/aidenlab/juicer) followed by ordering and orientation via 3D-DNA v.180922 (https://github.com/aidenlab/3d-dna)^61–63^. All scaffolded assemblies then underwent manual curation using Juicebox v.1.11.08 (https://github.com/aidenlab/Juicebox)^61^.

### Assessing genome content

Genome assembly completeness was assessed using BUSCO v.5.8.2 (https://gitlab.com/ezlab/busco) with the viridiplantae_odb10 database^64^. To estimate genome sizes, we counted *K*-mers derived from Illumina short reads using Jellyfish (https://github.com/gmarcais/Jellyfish)^65^. The clonality of the hybrid was also assessed using *K*-mers within a dataset of 12 samples. Meryl v.1.4.1 (https://github.com/marbl/meryl) with meryl_dif_history computed shared and unique *K*-mers for all representative sample pairs^66^. We annotated gene encoding sequences in the scaffolded assemblies (Zmar913-Mar3, Zpac1022-Sio2 and both haplotypes of hybrid Zmar912-Mar1) using gene models produced by Helixer v.0.3.3 (https://github.com/weberlab-hhu/Helixer), a deep neural network gene prediction tool^67^. Using the land_plant database, we found that Helixer performed better for BUSCO completeness metrics than cDNA-informed gene calls. For long terminal repeat/transposable element content, we annotated repeats using EDTA v.2.2.0 (https://github.com/oushujun/EDTA) running RepeatModeler (sensitive), excluding un-scaffolded contigs from this analysis^68^.

### Speciation divergence time estimation and phylogenetic reconstruction

OrthoFinder v.3.0 (https://github.com/davidemms/OrthoFinder) used 104 orthogroups representing conserved single-copy genes to construct a multiple sequence alignment (MSA) and species tree for the plant proteomes specified in Supplementary Table 1 (refs. ^16,17,69–73^). From this MSA and tree topology, we extracted all monocot species, excluding *Triticum aestivum* due to alignment gaps, and for *Zostera* spp. included only *Zostera_marina_913* and *Zostera_pacifica_1022* to reduce redundancy. MCMCtree within PAML v.4.9 (https://github.com/abacus-gene/paml) was used for phylogenetic reconstruction with this protein MSA as input and calibration points of 42–52 Ma for *Oryza sativa* / *Brachypodium distachion* divergence, 118–129 Ma for *Spirodela polyrhiza* / *Z. marina* and 130–140 Ma for aquatic monocots, with hard bounds on tail probabilities^16,74^.

The resulting input species tree with 21 species was as follows: (Acorus_americanus, (((Colocasia_esculenta, (Spirodela_polyrhiza, Lemna_minor)), (Thalassia_testudinum, ((Cymodocea_nodosa, Posidonia_oceanica), (Potamogeton_acutifolius, (Phyllospadix_torreyi, (Zostera_marina_913, Zostera_pacifica_1022))))))‘B(1.18,1.29,1e-300,1e-300)’, (Dioscorea_dumetorum, ((Ananas_cosmosus, (((Zea_mays, Sorghum_bicolor), Oropetium_thomaeum), (Oryza_sativa, (Phyllostachys_edulis, Brachypodium_distachyon))‘B(0.42,0.52,1e-300,1e-300)’)), (Asparagus_officinalis, Phalaenopsis_equestris))))‘B(1.3,1.4,1e-300,1e-300)’).

MCMCtree was run with independent rates using an approximate likelihood calculation. Root age was constrained to <200 Ma, and the rate of 6.5 × 10^−9^ substitutions per site from *Oryza sativa* informed an rgene_gamma of 65 100, where 65/100 = 0.65 substitutions per site per time unit of 100 Ma (ref. ^75^). After a burn-in of 10,000, 200,000 samples were taken with a sampling frequency of 10 for a total of 20,000 samples, and a second independent run with the same parameters proved model convergence with an *R*^2^ of 0.9998. Acceptance proportions of node ages remained stable throughout both runs between 0.20 and 0.40, indicating sufficient burn-in and adequate priors. All node ages fall within the 95% confidence intervals of the phylogeny reported in Ma et al.^16^.

In parallel, we calculated the divergence time between *Z. marina* and *Z. pacifica* using genome-wide *K*_a_/*K*_s_ ratios and the same 6.5 × 10^−9^ synonymous substitution rate from *O. sativa* ^75^. MCscan Python within JCVI-toolkit v.1.5.3 (https://github.com/tanghaibao/jcvi) was used to compute syntenic orthologues from gene annotation coding sequences and proteins, and these gene pairs were supplied to gKaKs v.1.3.0, a pipeline validated on closely related species^76,77^.

### Pangenome

All *Z. marina* and *Z. pacifica* assemblies derived in this study, as well as the v.3.1 *Z. marina* reference genome (Zmar668), were fed into PanKmer (https://salk-tm.gitlab.io/pankmer/) genome-coverage to create a *Zostera* pangenome *K*-mer index^16,78^. Individual indices were produced for *Z. marina* and *Z. pacifica* genomes only, and these species-specific *K*-mer pools were used to compute percentage *K*-mer conservation across all hybrid chromosomes via the anchorplot functionality.

To plot chromosome 1 synteny (Fig. 2c) across the pangenome, we first scaffolded the draft genomes using our chromosome-resolved assemblies. Scaffolding used RagTag v.2.1.0 (https://github.com/malonge/RagTag) with the Zmar913-Mar3 assembly as a reference for all *Z. marina* and Zpac1022-Sio2 for all *Z. pacifica*^79^. Helixer v.0.3.2 produced gene models for the scaffolded assemblies, and we aligned chromosome 1 annotations with GENESPACE v.1.3.1 (https://github.com/jtlovell/GENESPACE) with the default parameters, specifying Zmar668 as the reference genome and a custom genome order for visualization using plot_riparian^80^.

We carried out all-by-all *K*_a_/*K*_s_ calculations of the California *Zostera* assemblies using MCscan Python with the JCVI toolkit v.0.9.14 (ref. ^77^). First syntenic orthologues were identified between the coding sequences of two genomes (jcvi.compara.catalog ortholog–no_strip_names). Next, the *K*_a_ and *K*_s_ values were estimated (jcvi.apps.ks calc–msa=muscle). The genomes described here were run all-by-all to determine all pairwise *K*_a_/*K*_s_ values, and the Yang–Nielson estimation was used for the reported data^81^.

### RNA-seq transcript mapping

The hybrid transcriptome used for RNA-seq mapping was derived from the Helixer-generated protein-coding gene annotations of the Hap1-*marina* and Hap2-*pacifica* assemblies, concatenated. Raw Illumina RNA-seq reads for all mesocosm experimental samples and the wild-collected *Z. pacifica* were mapped to the hybrid transcriptome using Salmon v.1.10.1 (https://github.com/COMBINE-lab/salmon)^82^. Next, we derived mapping accuracy for all genes with orthologues between *Z. marina* and *Z. pacifica*. We ran OrthoFinder v.3.0 on a set of 29 plant proteomes presented in Supplementary Table 1, including hybrid Hap1-*marina* and Hap2-*pacifica*, and extracted all one-to-one gene pairs, excluding one-to-many pairings^69^. A set of 22 *Z. marina* RNA-seq samples (all mesocosm samples) and 7 *Z. pacifica* samples were used to derive mapping accuracy for each gene pair. We defined mapping accuracy as the percentage of expected mapping (*Z. marina* transcript to Hap1-*marina*, or *Z. pacifica* transcript to Hap1-*pacifica*) out of all mapping in terms of read counts and derived the median accuracy within the sample set for each gene to reduce outlier effects from samples with very low mapping. Supplementary Data 3 contains the gene pairs with accuracy >90% that were carried forward to differential expression analysis.

### Differential expression and GO enrichment

To isolate Hap1-*marina* and Hap2-*pacifica* transcription for direct comparison of 1:1 orthologues, each hybrid sample was split post-mapping into two subsamples (Hap1-*marina* and Hap2-*pacifica*), and gene IDs were normalized to Hap1-*marina*. Differential expression was carried out using a Python implementation of DESeq2, PyDESeq2 v.0.4.12 with default_inference (https://github.com/owkin/PyDESeq2), using salmon read counts (NumReads) as input^83^. These quants data are presented in Supplementary Data 4. DE gene significance was defined by a DESeq2 adjusted *P* ≤ 0.05 across replicates and a log_2_ fold change absolute value ≥0.5. Biased expression is defined as DE genes between the Hap1-*marina* and Hap2-*pacifica* subsamples. For the comparison of full-light transcription (t1 and t4) to reduced light (t2 and t3), we aggregated the reduced-light samples as replicates to remedy replicate loss for *Z. marina*, as detailed in Supplementary Table 3. For each tissue and accession, we derived enrichment for all DE genes displaying upregulation in the low-light treatment ((t2 + t3):t1 and (t2 + t3):t4) and downregulation (t1:(t2 + t3) and t4:(t2 + t3)), to extract genes that were differentially expressed in the shade time points as compared with either t1 (control) or t4 (recovery). We treated the hybrid and *Z. marina* as distinct species and therefore analysed them via PyDESeq2 as separate datasets, compared through GO term enrichment analysis. For GO term enrichment analysis of each tissue and accession, we used GOATOOLS v.1.4.12 (https://github.com/tanghaibao/goatools) with the go-basic library and fdr_bh multiple test correction^84^. Exhaustive results of the GO enrichment analysis are presented in Supplementary Data 5.

### Plastid relative coverage

Known *Z. marina* chloroplast and mitochondrion complete genomes—accessions NC_036014.1 and NC_035345.1, respectively—were concatenated to the chromosomal sequences of the Zmar912-Mar1 hybrid. Illumina WGS reads from the 12 tank samples were mapped to the combined sequences using minimap2 v.2.24-r1122 (https://github.com/lh3/minimap2) with secondary mapping disabled^85^. Bedtools v.2.30.0 (https://github.com/arq5x/bedtools2) was then used to obtain the coverage across the sequences, and the median read coverage of each sequence was recorded for all samples^86^. The median read coverages for the two plastid sequences were divided by the average chromosome mean read coverage to compare changes in relative plastid number for each time point and are presented in Extended Data Fig. 8.

### Reporting summary

Further information on research design is available in the Nature Portfolio Reporting Summary linked to this article.

## Supplementary information

Reporting Summary

Supplementary Tables Supplementary Tables 1–5.

Supplementary Data Supplementary Data 1–5.

## Data Availability

All sequencing reads have been deposited in the NCBI Sequence Read Archive under BioProject accession PRJNA1237350. The assemblies are available via Figshare, with *Z. marina* at 10.6084/m9.figshare.28702679 (ref. ^87^), *Z. pacifica* at 10.6084/m9.figshare.28710650 (ref. ^88^) and hybrid *Z. marina* × *pacifica* at 10.6084/m9.figshare.28710710 (ref. ^89^).
