# Supplementary figures and images for: Hybridization and low-light adaptability in California eelgrass (Zostera spp.)
Source: Nat Plants. 2025 Oct 29;11(11):2409–22. doi: 10.1038/s41477-025-02142-2 (PMC12626889; doi:10.1038/s41477-025-02142-2)

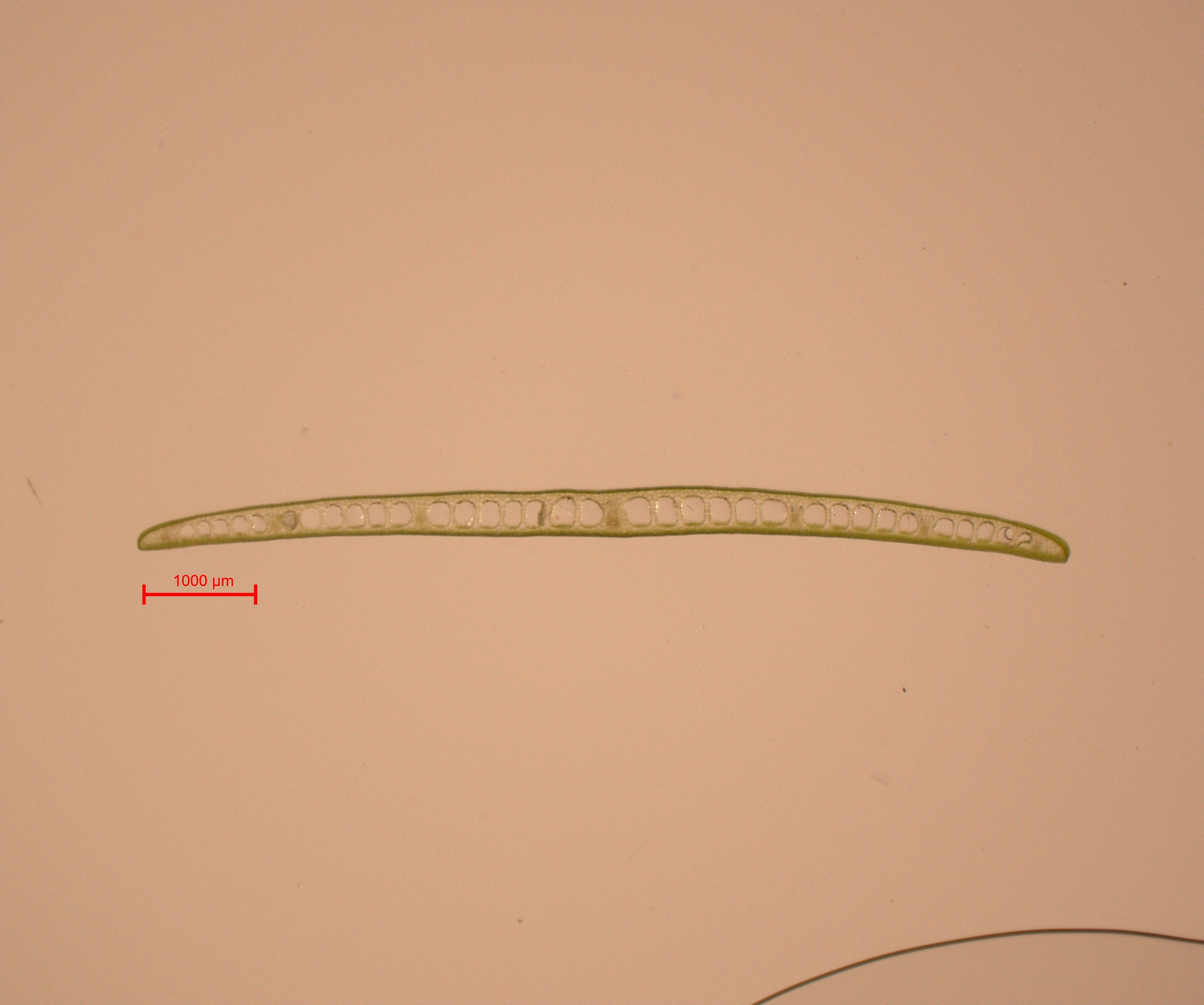

Supplement: Supplementary file 3 — Supplementary Data 1–5. [file 41477_2025_2142_MOESM3_ESM.zip › supplementary_data/Supp_data_1_images/hybrid.jpg]

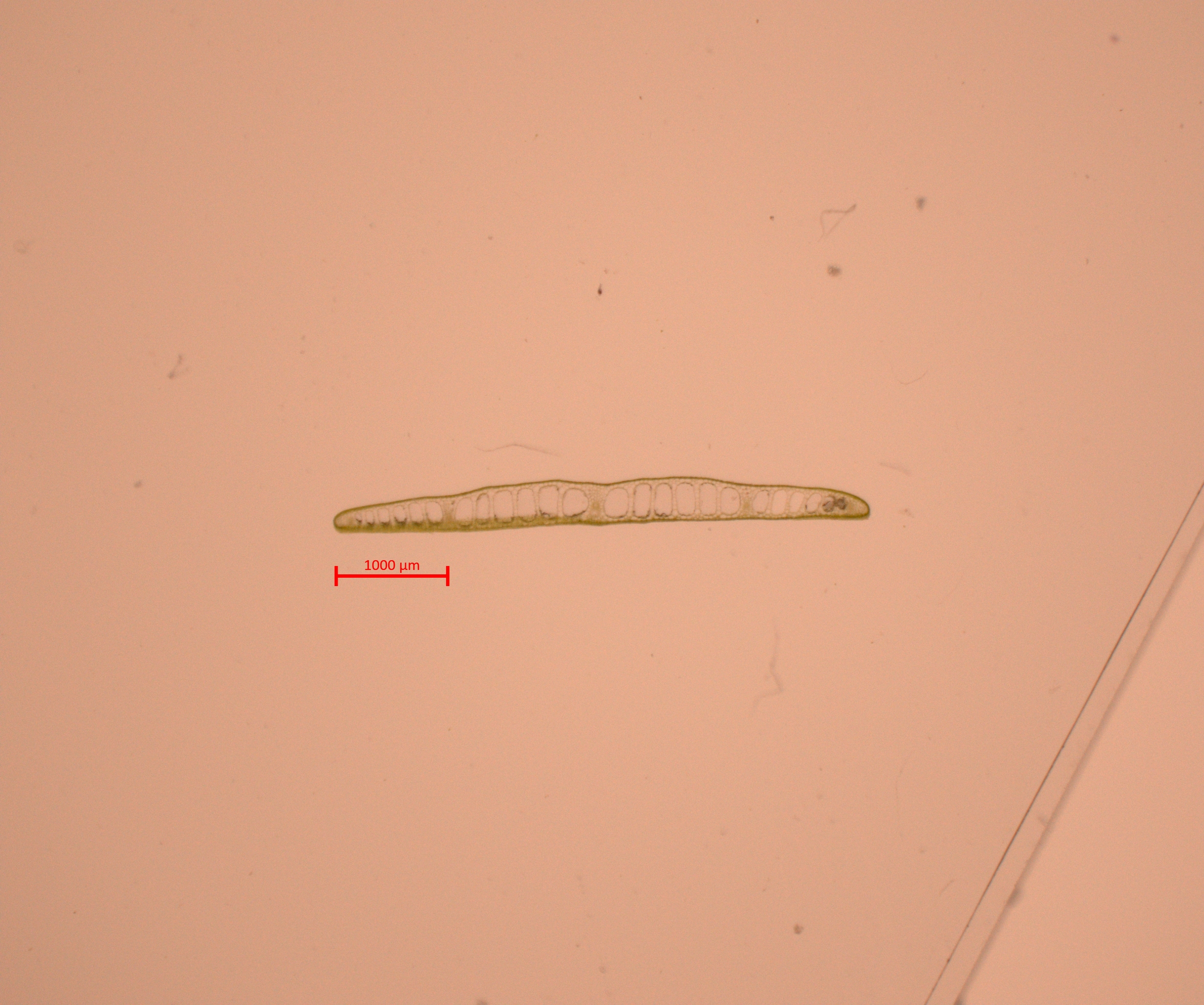

Supplement: Supplementary file 3 — Supplementary Data 1–5. [file 41477_2025_2142_MOESM3_ESM.zip › supplementary_data/Supp_data_1_images/marina.jpg]

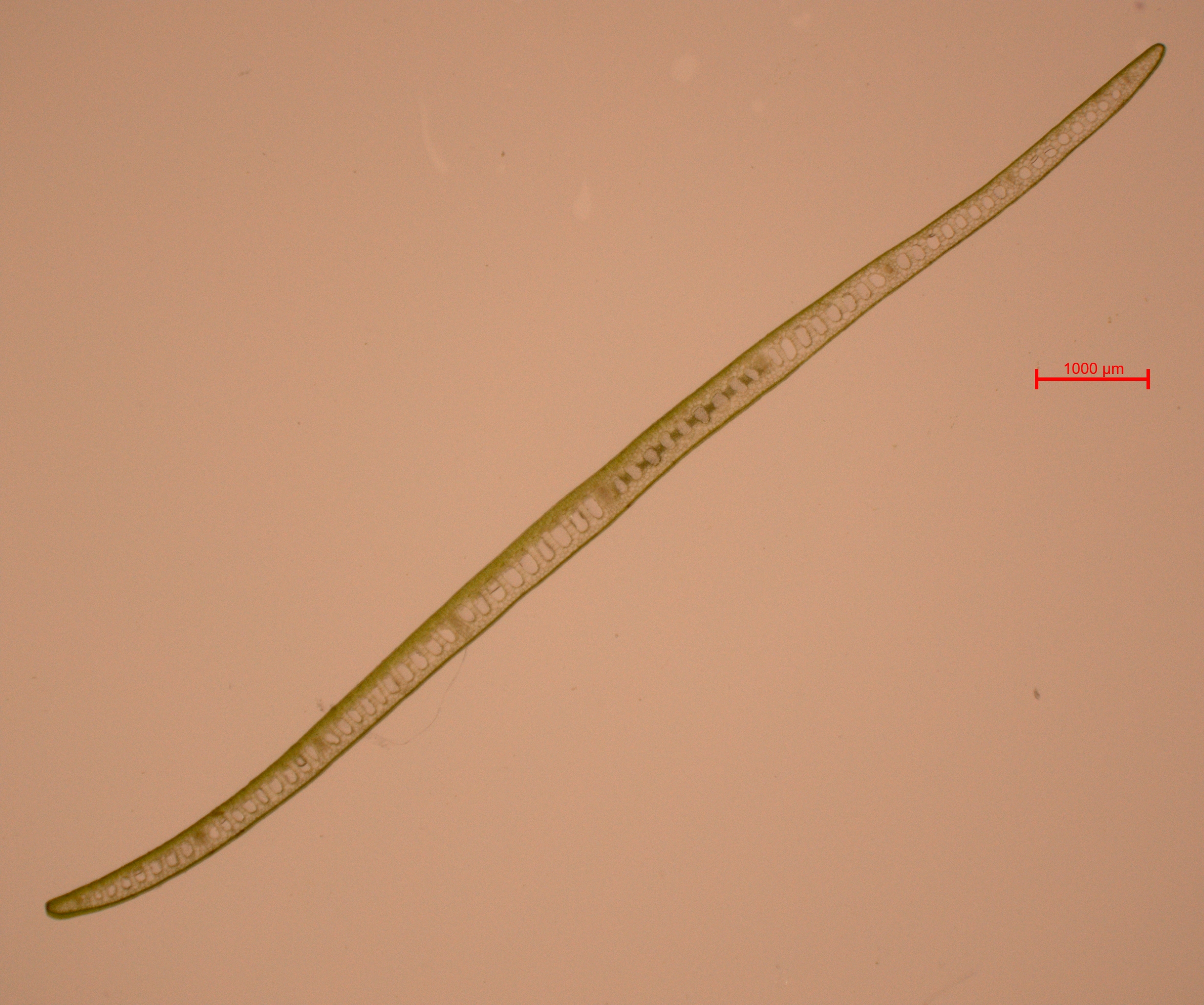

Supplement: Supplementary file 3 — Supplementary Data 1–5. [file 41477_2025_2142_MOESM3_ESM.zip › supplementary_data/Supp_data_1_images/pacifica.jpg]
